# Can we predict severity of acute cholecystitis at admission?

**DOI:** 10.12669/pjms.345.14502

**Published:** 2018

**Authors:** Sadettin Er, Sabri Ozden, Canbert Celik, Bulent Cavit Yuksel

**Affiliations:** 1 Sadettin Er, MD. Department of Surgery, Ankara Numune Education and Research Hospital, Ankara, Turkey; 2 Sabri Ozden, MD. Department of Surgery, Ankara Numune Education and Research Hospital, Ankara, Turkey; 3 Canbert Celik, MD. Department of Surgery, Ankara Numune Education and Research Hospital, Ankara, Turkey; 4 Bulent Cavit Yuksel, MD. Associate Professor Department of Surgery, Ankara Numune Education and Research Hospital, Ankara, Turkey

**Keywords:** Acute cholecystitis, Tokyo Guidelines

## Abstract

**Background and Objective:**

Acute cholecystitis (AC) is an inflammation of the gallbladder. Tokyo Guidelines (TGs) for the diagnosis of AC classified this condition according to severity as mild, moderate and severe. Therapeutic intervention regulated according to the type of severity. This study aimed to determine laboratory parameters that predict the severity of AC at hospital admission.

**Methods:**

One-hundred and ten patients with AC were retrospectively reviewed. These patients were treated in our department of surgery within a one-year period (2015–2016). Three patient groups were formed depending on the severity of Acute cholecystitis.

**Results:**

The baseline mean values for white blood cell count (WBC), blood urea nitrogen (BUN), creatinine and international normalized ratio (INR) were higher in the severe patient group at a significant level compared to the mild patient group. The WBC level was also significantly higher in the moderate group than the mild group. However, none of the laboratory parameters differentiated the severe group from the moderate group.

**Conclusion:**

Acute cholecystitis patients with high WBC, BUN, creatinine and INR levels at admission should be referred to an advanced care center for management.

## INTRODUCTION

It is estimated that in Western countries, inflammation of gallbladder is seen at a rate of 10% to 15%, and this prevalence increases by age. The majority of the patients are asymptomatic, and approximately 20% of symptomatic patients present with acute cholecystitis (AC).^1^

Acute cholecystitis presents with episodic pain in the epigastrium or right upper quadrant (RUQ) of the abdomen. Sometimes, it is difficult to distinguish AC from biliary colic. To set the diagnostic criteria for this condition based on clinical and biochemical parameters and imaging results, Tokyo Guidelines were published in 2007 (TG07).^2^,^3^ In 2013, these guidelines were updated in order to further increase the accuracy of diagnosis (TG13), and it has been reported that the accuracy rate using TG13 is greater than 90%.^3^ The last version of the guidelines were presented in 2017 and are known as TG18.^4^ According to TG13 and TG18, AC is graded as mild, moderate or severe based on the severity of findings. In moderate and severe forms, inflammation is more prominent. The severity of AC can be determined after a minimum of 72 hours of hospitalization. intervention is regulated according to this severity.^3^ Prediction of severity at hospital admission can help the surgeon decide on an appropriate therapy. Therefore, in the current study, we aimed to determine laboratory parameters that predict the severity of AC at admission.

## METHODS

The data were collected from patients with AC treated in our department of surgery within a one-year period (2015–2016) and were retrospectively reviewed. The baseline data, including age, sex, and medical comorbidities as defined by the American Society of Anesthesiologists (ASA) were retrieved for each patient.

We retrospectively examined the patients’ computer records to obtain data concerning medical history, physical findings, computerized tomography and ultrasound images, and laboratory tests undertaken at admission. All this information was used to evaluate the severity of AC according to the criteria of TG18. Based on the severity of AC, three patient groups were formed: mild, moderate, and severe.

In our department, the routine procedure for all AC patients is to start combined intravenous antibiotics, which generally contain both metronidazole and cephalosporin. Only the patients who underwent surgery within 72 hours of the onset of symptoms were included in the study. Surgical/anesthesiology information forms were checked to obtain detailed data on the surgical procedure. In addition, complications and the length of hospital stay were retrieved from hospital discharge notes.

The data were statistically analyzed using SPSS v. 22 (IBM Corp). The continuous variables were presented in number and percentages, and central tendencies were expressed with the mean plus standard deviation. The statistical significance of the values was determined according to the results of the chi-square analysis at a plevel of <0.05. In addition, one-way ANOVA was used to perform multiple comparisons in order to determine the significance of differences between the three groups. The groups that were found to have significant differences were further investigated using the Bonferroni correction.

### Ethical Committee

The approval of the ethical committee of Ankara Numune Training and Research Hospital was obtained prior to the study (Approval no: E-17-1509).

## RESULTS

A total of 110 patients with complete charts were included in the study. Eighty-four patients were male, and 26 were female, with a median age of 54 years (23 - 89). The severity grade was I (mild) in 62.7% of the patients, II (moderate) in 23.6%, and III (severe) in 13.6% (Table-I).

**Table-I:** Grading of AC severity according to TG18.

Grading of severity	n (%)
Grade I (mild)	69 (62.7%)
Grade II (moderate)	26 (23.6%)
Grade III (severe)	15 (13.6%)

AC: acute cholecystitis; TG18: Tokyo Guidelines 2018

Laparoscopic cholecystectomy (LC) was performed on 73 cases (66.4%), and open cholecystectomy (OC) on 37 patients (33.6%). LC was undertaken for 54 patients (78.2%) in the mild group. OC was performed on 14 patients (93.3%) in the severe group and 15 patients (21.7%) in the mild group. According to the final pathology reports, two of the 15 patients with severe AC had adenocarcinoma and the remaining 13 had necrotizing cholecystitis.

The multiple one-way ANOVA including Bonferroni’s correction revealed that the mean values of white blood cell (WBC) count, blood urea nitrogen (BUN), creatinine, and international normalized ratio (INR) were higher in the severe group at a significant level compared to the mild group. The WBC level was also significantly higher in the moderate group compared to the mild group. However, none of the laboratory parameters were able to differentiate the severe form of AC from the moderate form. Table-II summarizes the findings obtained.

**Table-II:** Levels of WBC, BUN, creatinine and INR according to AC severity.

Severity	WBC (×10^9^/L)	BUN (mg/mL)	Creatinine (mg/mL)	INR
Grade I (mild)	11.1±3.2	29.5±13.7	0.9±0.2	1.12±0.1
Grade II (moderate)	16.6±5.9	39.3±19.1	1.03±0.1	1.23±0.1
Grade III (severe)	17.5±8.3	47.3±30.04	1.3±0.8	2.02±0.8

WBC: white blood cell count; BUN: blood urea nitrogen; INR: international normalized ratio; AC: acute cholecystitis. The values are expressed as mean ± standard deviation.

## DISCUSSION

TG07, TG13 and TG18 provide simple criteria that not only facilitate the diagnostic process but also allow classifying the severity of AC.^2^-^4^ Prediction of severity at hospital admission can assist the clinician in properly managing the AC cases. Previous research showed that the requirement of intensive care after surgery was seen in more than 20% of the patients having severe inflammation of gallbladder.^5^ In another study, Gurbulak et al. demonstrated that C-reactive protein can be considered as a strong predictor of different AC grades according to TG13, and treatment can be reliably planned according to this classification.^6^ Our findings showed that the WBC count differentiated between mild and moderate AC groups, which is in agreement with the results of Gurbulak et al.^6^ Furthermore, in the current study, WBC, BUN, creatinine, and INR values were found valuable in distinguishing the mild and severe forms of the disease. To the best of our knowledge, this finding has not been reported in the medical literature before.

In different studies conducted using TG07, the proportion of the severe AC patients ranged from 1.2% to 6%.^7^,^8^ A recent study conducted by Yokoe et al. reported the severe AC ratio as 17.2% according to TG13.^9^ In the current study, the severe AC ratio was 13.6%. All these results indicate that the prevalence of the severe form of AC is gradually increasing.

TG13 and TG18 describe the severe form of AC as associated with dysfunctions in the organ system that may sometimes require intensive care.^4^,^10^,^11^ Gonzalez-Munoz et al. performed a logistic regression analysis to predict of the prognosis for AC and found TG13 severity grading as a predictor for mortality at hospital admission.^10^ In contrast, Yokoe et al. reported the rate of mortality in AC as only 1%.^9^ Other researchers showed that multivariate analysis revealed TG13 and TG18 grading as an independent predicting factor for the length of hospital stay and requirement for OC.^4^,^12^ Similarly, in their study including cases with intraoperative bile duct injury, Tornqvist et al. identified complications at a significantly higher rate in patients with a more severe form of AC.^13^ All these studies confirm that an increase in the number of severe (Grade III) AC patients affects complications and surgical planning, which naturally leads to increased morbidity and mortality rates.

The patients with mild AC can be considered as candidates for LC, those in the moderate group can be treated with LC in equipped hospitals or managed with percutaneous cholecystostomy, and those having severe AC require percutaneous cholecystostomy.^14^ The difficulties involved in the surgery of cases having a severe form of AC vary according to the state of inflammation and fibrosis. For example, bile duct injury is among the factors that increases the grading of AC, thus the difficulty of surgery. This is also the reason for performing LC on severe AC cases only after draining the gallbladder. Ambe et al. used a multivariate analysis and determined the following eight factors as being independently associated with AC and increasing its severity: gender, age, body mass index, ASA score, recurrent colic, thickness of the gallbladder wall, WBC count, and CRP value. The authors scored each of these factors from 1 to 9, with a score of 7 or greater predicting patients with severe AC. The scoring presented in the study was reported to be associated with operating time, intensive care requirement, and length of hospital stay; however, it was not related to complication rate or the rate of conversion to open surgery.^15^ Similarly, Borzellino et al., described the criteria for the differential diagnosis of gangrenous and phlegmonous cholecystitis in AC cases. The authors identified the following four factors as being independent predictors for the higher grading of AC: fever (38 °C), gallbladder distension, edema of the gallbladder wall, and preoperative adverse events.^16^ In this study, using simple laboratory parameters that predicted the severity at admission, especially in primary / secondary care centers, the patient was directed to a higher center due to the need for endoscopic intervention by an experienced team or intensive care. Our study partially showed that simple laboratory parameters can predict the severity at admission and by this way primary/secondary care centers can refer the patient to higher center for endoscopic intervention, or experienced surgeon, or intensive care. We consider that OC should be performed particularly in cases that have a risk of bile duct injury or damage to blood vessels to prevent perioperative or postoperative complications.

### Limitation of the study

One limitation of this study is that it was undertaken retrospectively. Thus, we suggest that the findings obtained from this study should be supported in further studies with a prospective design.

### Authors’ Contribution

**SE:** Data collection, study design, manuscript writing, final manuscript approval.

**SÖ:** Data collection, study design, manuscript drafting, data analysis, manuscript approval.

**CÇ:** Data collection, manuscript approval and data interpretation.

**BCY:** Data collection, writing, revise, editing and final manuscript approval.
